# Artificial Intelligence and Surgical Education in the UK: A Systematic Review of Current Use, Evidence Gaps and Future Directions

**DOI:** 10.7759/cureus.98231

**Published:** 2025-12-01

**Authors:** Ranj Bhakar, James Miller, Amelia Simenacz

**Affiliations:** 1 Trauma and Orthopaedics, Torbay Hospital, Torquay, GBR; 2 Trauma and Orthopaedics, Torbay and South Devon NHS Trust, Exeter, GBR; 3 Urology, Royal Free Hospital, London, GBR

**Keywords:** ai, artificial intelligence, machine learning, medical education, surgical education, surgical training, united kingdom

## Abstract

Artificial intelligence (AI) offers new opportunities to enhance surgical training through automated performance assessment, adaptive learning platforms, and AI-enabled virtual or augmented reality (VR/AR) simulation. Although global literature is expanding, the UK context differs in governance, procurement, and training structures. National initiatives, such as the Royal College of Surgeons of England (RCS) Future of Surgery (FOS) programme, have highlighted AI and extended reality as priorities for modernising surgical education. However, UK peer-reviewed evidence remains limited, with most work consisting of pilot studies and early feasibility assessments. This narrative review synthesises UK-specific applications of AI in surgical training, identifies current gaps, and proposes priorities for future research.

Two independent reviewers conducted a focused search of peer-reviewed and grey literature, including RCS policy documents, to identify UK-based uses of AI in surgical training. Databases searched included PubMed, the Excerpta Medica database (Embase), Scopus, and Web of Science, supplemented by targeted screening of UK policy sources. Studies were included if they involved AI or AI-enabled technologies applied to surgical education, simulation, or assessment within the UK. Non-UK studies and articles focused solely on clinical (non-educational) AI applications were excluded. Data were synthesised on AI modality, educational outcomes, feasibility, and barriers. A Risk Of Bias In Non-randomized Studies - of Interventions (ROBINS-I)-aligned risk-of-bias assessment was performed.

Results: UK literature comprises national policy reports and a small number of empirical pilot studies exploring AI-enhanced VR/AR simulation, AI-driven performance analytics, and early AI components within robotic training curricula. RCS policy documents consistently identify AI as a key element of future training reform. Empirical studies report feasibility, trainee acceptability, and construct validity but provide limited evidence of improvements in operative performance or patient outcomes. Most work is single-centre and exploratory, and significant barriers, including cost, faculty training, data-governance requirements, and variability in access across deaneries, remain.

Discussion: The UK is at an early yet promising stage of adopting AI within surgical education. National policy momentum and the expansion of robotic programmes provide opportunities for coordinated integration. Collaboration between NHS education bodies, simulation centres, and technology developers could support standardised metrics and equitable access. However, robust multi-centre evaluation frameworks are required to determine educational effectiveness. Ethical considerations, including data privacy, algorithmic transparency, and the impact of automated feedback on trainee development, require careful attention.

Conclusions: AI use in UK surgical training is emerging but currently driven largely by pilot studies and policy direction rather than high-quality outcome evidence. Major gaps include multi-centre validation, curriculum integration, standardised assessment frameworks, and equitable access to AI-enabled systems. Future UK research should prioritise structured validation studies and national coordination to define effective, scalable, and safe AI tools that can enhance surgical education across the NHS.

## Introduction and background

Artificial intelligence (AI) is fast-moving, evolving and expanding in almost every aspect of daily living; it is also furthering its expansion with its use in the healthcare setting [1]. However, in the context of medical education, it is still in its infancy. AI being used globally in the context of surgical training is steadily increasing, with the majority of evidence of its use coming from the United States of America; however, little evidence exists in regard to its use in the UK [2].

AI has the capacity to analyse a high volume of performance data, allow for automated feedback, direct feedback in real time, and then subsequently tailor training experiences through adaptive learning. The capabilities of AI have the potential to enhance traditional teaching models, such as at cadaver teaching days, and accelerate skill acquisition. Across the world, AI has been further integrated into virtual reality (VR) and augmented reality (AR) within simulators, robotic training systems and automated skill-assessment platforms.

Within the UK, surgical education is consistently delivered within a highly structured postgraduate framework under the Intercollegiate Surgical Curriculum Programme (ISCP), which is regulated by the overarching governing body, the General Medical Council (GMC). Although the ISCP portfolio allows some workplace-based assessments to be submitted under a simulated environment, it has no formal integration of AI into its structure. This existing paradigm in the UK offers an exciting environment for the deployment of AI, in particular due to the emphasis on competency-based progression and objective assessment. Despite this, UK-specific evidence of AI use within formal surgical training remains limited in terms of quantity of evidence and quality of evidence beyond pilot studies.

This narrative review aims to synthesise the published and grey literature describing the use of AI in surgical training within the UK, such as machine learning (ML) and AI-assisted AR/VR tools. With the aims of identifying active areas of implementation, highlighting any key barriers to its expansion and defining future priorities for further integration.

## Review

Methods

**Figure 1:**
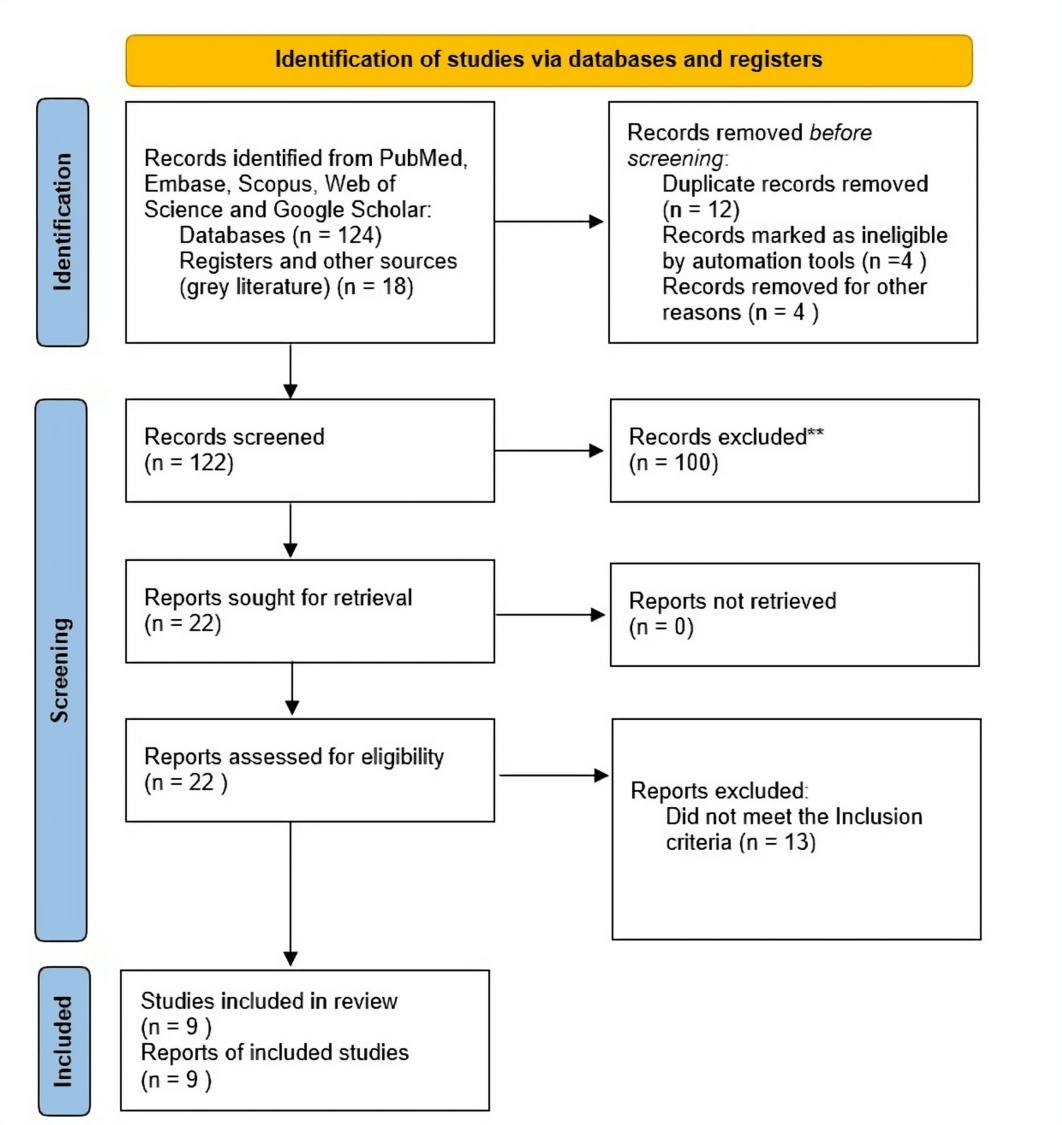
PRISMA flow diagram PRISMA: Preferred Reporting Items for Systematic Reviews and Meta-Analyses; Embase: Excerpta Medica database

A focused literature search of English-written studies was conducted across multiple publication libraries, including PubMed, the Excerpta Medica database (Embase), Scopus, Web of Science and Google Scholar, searching for any publications up to October 2025. Screening followed Preferred Reporting Items for Systematic Reviews and Meta-Analyses (PRISMA) 2020 guidelines (Figure 1). The following search terms were used and were adjusted for differing indexing systems, allowing us to reach as many studies that were likely to be relevant for UK studies: “Artificial intelligence” OR “machine learning” OR “deep learning” OR “virtual reality” OR “augmented reality” AND (“surgical training” OR “surgical education”) AND (“United Kingdom” OR “NHS” OR “Royal College of Surgeons”.

Two reviewers independently screened titles and abstracts, retrieved full texts of potentially eligible articles, and extracted data using a pre-specified form addressing AI technology tool type, training setting, study type, participants, and outcomes such as AI tool feasibility and educational effect, in addition to any barriers that were found in the implementation of the AI tool. Any disputes were discussed and settled with a third independent reviewer.

Policy documents directly from the Royal College of Surgeons of England (RCS) were also reviewed.

The inclusion criteria included any study or report conducted in the UK, as well as the use of AI or AI-enabled technologies for surgical training, simulation or assessment.

Any study focusing solely on the clinical use of AI, such as diagnostic or predictive AI, without an educational component, was excluded. Any study containing a mix of elements was included as long as there was an educational component of the AI tool. In addition, any non-UK studies were also excluded, as the aim of the review is to investigate the current use and potential further application of AI in current UK healthcare systems and training curricula, which differ significantly from other global healthcare systems.

The relevant studies were analysed, and data were extracted, including publication type, the type of AI modality, outcomes and key findings. The results were grouped by application domain.

Risk of Bias Assessment

The methodological quality of included studies was assessed using a simplified version of the Risk of Bias in Non-randomised Studies of Interventions (ROBINS-I) approach, adapted for pilot, qualitative and policy research. This particular tool was chosen as it provides a systematic way of evaluating a study’s internal validity, in addition to providing a clear way of understanding a study’s weaknesses and strengths. Each study was evaluated across domains, including confounding bias, measurement bias, and reporting bias. Ratings were then classified as low, moderate, moderate-high and high risk of bias (Table 1).

**Table 1:** Risk of bias assessment of the selected studies RCS: Royal College of Surgeons of England; AR: augmented reality; VR: virtual reality

Study (year)	Study type	Bias due to confounding/selection	Bias in the measurement of outcomes	Bias due to missing data/reporting	Overall risk of bias
RCS England (2022): Future of Surgery report [3]	Policy/report	Low (expert-driven, not data-based)	Moderate (narrative, no measurable outcomes)	Low (publicly available, transparent)	Moderate
RCS England (2025): Technology Enhanced Surgical Training (TEST) [4]	Policy/report	Low	Moderate (expert opinion without data validation)	Low	Moderate
Ogbonnaya et al. (2025) [5]	Experimental/AI suturing	Low–Moderate (clear algorithm validation but small cohort)	Low (objective motion-tracking data)	Low	Low–Moderate
Colman et al. (2025) [6]	Pilot study (AR)	Moderate (small sample, no control)	Moderate (self-reported engagement measures)	Moderate (limited outcome detail)	Moderate–High
Please et al. (2024) [7]	Qualitative (VR)	Moderate (volunteer bias possible)	Moderate (subjective perceptions)	Moderate	Moderate–High
Al-Ani et al. (2025) [8]	Pilot (robotic training)	Moderate (non-randomised, single-centre)	Moderate (subjective trainee evaluation)	Moderate	Moderate–High
Harris et al. (2025) [9]	Consensus/Delphi	Low (diverse expert group, clear methodology)	Moderate (subjective expert opinion)	Low	Moderate
Al-Saadawi et al. (2025) [10]	Narrative review (orthopaedics)	Moderate (selection bias from limited inclusion criteria)	High (heterogeneous data, narrative synthesis)	Moderate	High
Anyinkeng et al. (2025) [11]	Systematic review (remote/virtual training)	Low–Moderate (transparent inclusion)	Moderate (self-reported data from included studies)	Low	Moderate

Limitations

This review was limited by the small volume of published UK-specific data, with most evidence derived from pilot studies and policy reports rather than randomised control trials (RCTs). This potentially limits the conclusions of this review, as more data and research are needed. In particular, RCTs on a national scale across multiple deaneries would supply excellent evidence on a national scale of the feasibility of the integration of AI into current UK training curricula. The search was restricted to English-language sources and, therefore, may have missed non-English-based technologies and studies, pilot studies, and review studies, and therefore may result in the lack of comprehensiveness of the review. With respect to the included studies in this review, due to their heterogeneous nature, a direct comparison between studies may not be feasible and may therefore potentially weaken the conclusions of this review.

Results

From the inclusion and exclusion criteria, nine UK-relevant publications were identified. These include four peer-reviewed empirical studies, three consensus or Delphi papers and two RCS policy documents from 2022 and 2025. It was noted that some studies specifically lacked the measurement of educational outcomes, such as skill retention and operative performance.

Summary of the Included Studies

A summary of the included studies is presented in Table 2.

**Table 2:** Summary of the selected studies RCS: Royal College of Surgeons of England; AR: augmented reality; VR: virtual reality

Author (year)	Study type	UK-based	Key study aim/Focus	Sample size (if reported)	AI/Digital modality	Main outcomes/Measures
RCS England (2022-2025 (report updates)) [3,4]	Policy/report (UK)	Yes	Technology-enhanced surgical training policy and strategic direction	Not applicable (policy report)	Broad AI, simulation, robotics, and digital training technologies	2022: recognised the upcoming need to integrate new innovations such as AI into training and within the workforce. 2025: growing use of AI/VR/AR systems and encouraged to be used by surgical teams.
Ogbonnaya et al. (2025) [5]	Systematic review (AI tools for suturing)	Includes UK examples	Review of AI-driven tools for laparoscopic suturing education, including UK data	Not applicable (systematic review)	AI models for error prediction and suturing performance during laparoscopy	The AI model predicted surgical errors during the training of robotic suturing. AI-driven tracking assessment for laparoscopic instrument feedback during training sessions.
Colman et al. (2025) [6]	Pilot study (AR)	Yes (multi-site UK)	Evaluation of the LapAR augmented reality device for laparoscopic surgical training	Not stated in the screening sheet (pilot trainees)	AI-augmented reality laparoscopic simulator with performance feedback	Immersive AI augmented reality systems have significantly improved the training of laparoscopic skills.
Please et al. (2024) [7]	Qualitative/implementation (VR)	Yes	Evaluation of VR technology for surgical learning and trainee experiences	Not stated in the screening sheet (trainees)	AI-enabled virtual reality platform for surgical skills training	Successfully demonstrated that VR can be used to upscale postgraduate surgical education, affirming its potential in healthcare capacity building throughout Africa, Europe and beyond.
Al-Ani et al. (2025) [8]	Pilot (robotic training)	Yes	Pilot programme for training trainees in robotic surgery in a UK setting	Not stated in the screening sheet (pilot trainees)	Robotic surgery training curriculum using simulation and digital assessment tools	A structured robotic training programme to democratise robotic surgical training and AI-assisted systems, especially among junior trainees who may not have prior laparoscopic surgical experience, is safe and feasible
Harris et al. (2025) [9]	Consensus/Study (UK)	Yes	Pan-speciality UK Delphi consensus on procedural robotic surgery training	Delphi panel (experts; consensus study)	Robotic training pathways; AI-informed metrics and curricula	Highlighted the feasibility and eagerness for AI and robotic surgery to be added to the curriculum for surgical training.
Al-Saadawi et al. (2025) [10]	Review (orthopaedics)	Includes UK examples	Review exploring current applications of AI in orthopaedic training (includes UK)	Not applicable (narrative/systematic review)	AI applications in orthopaedic training and simulation	AI holds major potential to revolutionise orthopaedic surgical training. However, evidence supporting its use in this field remains limited
Anyinkeng et al. (2025) [11]	Review (digital modalities)	Includes UK examples	Review of remote and virtual surgical training with examples, including the UK	Not applicable (review)	Remote, virtual and digital surgical training platforms (some AI-enabled)	pinpoints the promise for strategic use of these digital training solutions, such as AI, in revolutionising cardiothoracic surgery training in low-resource settings, whilst maintaining high standards for training.

The RCS Future of Surgery (FOS; 2025) and Technology Enhanced Surgical Training (2022) reports outlined the college’s national priorities for integrating simulation, robotics, and AI into surgical training [3, 4]. These RCS documents highlight the potential of AI-driven analytics for objective performance feedback and increasing equitable trainee access through virtual platforms.

UK pilot studies have explored AI-driven assessment in laparoscopic and robotic surgery. Ogbonnaya et al.’s review showed how an AI-based suturing education system using motion tracking is being used to predict performance grades, showing a strong correlation with expert-level assessment [5]. This was done using motion capture, deep AI learning and video segmentation with real-time feedback to improve learner skill. Similarly, Colman et al. evaluated an AR-assisted laparoscopic trainer, demonstrating its feasibility and positive trainee perception [6]. This study measured procedure completion time as an objective performance measure in addition to an in-depth interview following the simulation.

Qualitative evidence by Please et al. reported increased engagement and perceived learning benefits from VR simulators incorporating AI feedback, although integration into the current surgical training curriculum remains limited [7].

A pilot robotic surgical training programme conducted by Al-Ani et al. amongst UK general surgical trainees showed that all participants demonstrated console-operative competency within a median of 4.5 days of AI/VR simulation training. This encouragingly demonstrates that early-stage robotic surgical training is feasible and well-received in a UK hospital setting [8]. It is noted that much of the pilot study data incorporates AI-assisted AR or VR technology within laparoscopic surgical training, potentially heavily suggesting that AI is being expansively integrated within general surgery rather than other surgical specialities such as vascular surgery.

A 2025 UK pan-speciality Delphi consensus defined the core competencies and evaluation metrics for robotic surgical training, advocating for AI-driven data analysis to standardise assessment [9]. Similarly, a review concerning AI use in orthopaedic training within the UK highlights the need for AI-assisted training in trauma and orthopaedics due to the competency-based procedural nature of orthopaedic skills. This would allow trainees to practise surgical skills in a risk-free environment in an effort to improve their competency [10].

Further evidence conducted by Anyinkeng et al. found that the UK is demonstrating that VR-augmented training is significantly improving learning rates amongst trainees. There was a 50% reduction in the learning curve for thoracoscopic lobectomy, highlighting its potential to accelerate skill acquisition [11].

Common barriers across the included literature include cost, inconsistencies across deaneries within the UK and limited faculty training opportunities. This was particularly echoed by trainees as reported by evidence supplied by Colman et al. and Al Ani [6, 8]. Results have also shown that strict NHS clinical governance requirements have and will likely continue to hinder AI dataset sharing, with the need for patient data being potentially kept stored on a private third-party server under the AI company. This was particularly emphasised by the RCS: FOS Policy documents [3,4]. This may also prevent the integration of these AI systems on a national scale, due to many hospitals having differing operating systems. No UK study to date has demonstrated significant improvement in operative outcomes directly attributable to AI-based training interventions; this is likely due to the lack of RCTs and longitudinal studies within the UK. Furthermore, not all studies included aimed at improvement in measurable operative outcomes, as Colman et al. [6], but rather focused on qualitative feedback from trainees, as evidence supplied by Please et al. has shown [7].

Discussion

This review has several important strengths. Firstly, it provides one of the few UK-focused analyses of AI use specifically towards surgical training, addressing a notable gap in the existing literature, where most published evidence originates from Asia or North America. With regard specifically to the NHS, RCS frameworks, and UK postgraduate training structures, this review offers contextualised insights that are directly relevant to national educational policy and curriculum design.

Second, the review incorporates a comprehensive search strategy using multiple major databases as well as grey literature, including RCS policy reports. The inclusion of grey literature is a particular strength because many UK developments in AI-enhanced training occur outside traditional academic publications, especially in pilot programmes, institutional evaluations, and consensus statements. This approach ensures a more complete understanding of AI use within the UK than would be possible with peer-reviewed studies alone.

Additionally, the review synthesises a diversity of study types, including pilot studies, qualitative work, consensus methodologies, national policy documents and narrative reviews. This further reflects the wide range of emerging AI applications within surgical education. This methodological breadth enables a broader perspective on how AI is conceptualised, implemented and evaluated across surgical training programmes.

This review demonstrates that AI in UK surgical education is at an early implementation stage. Evidence supports the feasibility of its use with growing trainee acceptance, but robust outcomes research is lacking. The UK benefits from national coordination between the NHS and RCS, which provides an infrastructure for standardised deployment and evaluation.

Evidence from the included studies supports the feasibility of AI-assisted training tools and indicates positive trainee acceptance within UK surgical education. Colman et al. [6] demonstrated that AI-integrated AR platforms were successfully implemented across multiple UK sites, with trainees consistently reporting that the system enhanced their understanding of procedural tasks and improved the usefulness of feedback. Similarly, Please et al. [7] identified strong trainee engagement with AI-enabled VR technology, highlighting themes such as improved confidence, clearer technical guidance, and enhanced motivation to practise.

Ogbonnaya et al. [5] further supported the feasibility by showing that AI-based motion-tracking systems could reliably generate performance assessments that aligned closely with expert opinion, suggesting AI tools are capable of providing meaningful and educationally valid feedback. Together, these studies illustrate that AI technologies can be feasibly integrated into UK training environments and are generally well received by trainees, who value the structured, objective and personalised nature of AI-generated feedback.

AI has proven to have potential in three main domains, which are as follows: (1) Objective skill assessment: AI systems use tools such as motion tracking, instrument trajectory analysis, and automated error detection to assess technical performance. Studies like Ogbonnaya et al. [5] show that ML models can generate objective ratings that closely align with expert evaluations, offering a consistent assessment of suturing and laparoscopic skills. These technologies support competency-based progression by reducing variability in trainer judgment. (2) Adaptive simulation (VR and AR): AI-enabled VR and AR platforms adjust task difficulty and feedback in real time according to trainee performance. Both Colman et al. [6] and Please et al. [7] describe systems that highlight errors, guide technique, and tailor scenarios to the learner’s ability. This creates personalised practice environments that support deliberate skill development, particularly valuable in a training system where operative exposure may vary between centres. (3) Robotic surgery training with AI analytics: As robotic surgery expands within the NHS, AI plays a growing role in analysing console movements, segmenting tasks, and identifying inefficiencies. The Harris et al. [9] consensus emphasises how these metrics help structure robotic curricula and provide transparent progression markers. Performance dashboards within robotic simulators allow trainees to monitor improvement and benchmark themselves objectively.

However, these AI tools remain fragmented, disjointed and largely pilot-based. Fragmentation is evident in the way AI tools are being developed separately across specialities and platforms. The AR laparoscopic trainer studied by Colman et al. [6], the AI suturing assessment described by Ogbonnaya et al. [5], and the VR systems examined by Please et al. [7] all function as standalone technologies with different metrics and feedback styles. Similarly, the robotic analytics discussed in Harris et al. [9] represent yet another isolated stream of development. Because these systems are not interoperable and use speciality-specific approaches, AI tools remain dispersed rather than part of a unified national framework, limiting standardisation and broader adoption.

Future progress will require multicentre validation studies, integration within the surgical portfolio ISCP and strong collaborations between surgeons, educators and technologists.

It is also prudent to address the ethical considerations introduced by AI in surgical training. Key issues include algorithmic transparency, potential bias in performance scoring, and the need to protect trainee autonomy. Within the UK, these concerns are closely aligned with emerging regulatory frameworks such as the NHS AI and Digital Regulations Service [12], which outlines requirements for data protection, model transparency, and clinical risk management, and the MHRA’s Software and AI as a Medical Device (SaMD/AIMD) guidance [13], which sets standards for validation, traceability, and safety monitoring. Aligning AI-based training tools with these frameworks will be essential to ensure responsible deployment within surgical education.

The UK therefore has an opportunity to lead internationally by developing a unified, regulation-compliant AI training framework and embedding data-driven feedback within ISCP portfolios. Future research should also explore how AI affects trainee autonomy, learning psychology, and feedback quality, alongside rigorous evaluation of outcomes such as skill retention and operative efficiency, to progress from pilot-stage feasibility to evidence-based policy integration.

Globally, several countries and professional bodies have taken a more coordinated approach to integrating AI-enabled simulation and robotic training into their surgical curricula. In the United States, the Society of Thoracic Surgeons (STS) has published an expert consensus framework outlining a standardised national robotic surgery curriculum, incorporating simulation, structured milestones and objective performance metrics as required components of trainee development [14]. Similar integration is seen across many US residency programmes, where AI-supported systems such as the da Vinci Skills Simulator and SimNow are embedded into stepwise training pathways before clinical console use. These models demonstrate how national endorsement, unified performance metrics and structured faculty involvement can accelerate the safe adoption of AI-enhanced training tools.

A 2023 systematic review by Satapathy et al. noted that over 70% of AI-enhanced surgical training studies originate from North America or Asia, highlighting the paucity of UK data [15]. This disparity underscores the importance of national coordination within the UK in fostering innovation within the NHS training system.

Compared with these examples, UK implementation remains more localised and pilot-driven, highlighting the need for coordinated national leadership through the NHS and RCS to achieve scalable and consistent adoption.

The aim of AI-integrated surgical training should be to advance surgical knowledge and increase the effectiveness of procedural skills. The integration of AI aligns well with educational theories such as deliberate practice and cognitive load theory, which enable repetitive, feedback-rich environments that reduce dependency on theatre time and expenses [16]. Adaptive AI algorithms can personalise task difficulty, maintaining trainees within their optimal learning zone. Such time-saving features could mitigate constraints posed by the European Working Time Directive (EWTD) and relieve pressures on operating theatres.

Deployment of AI-enabled simulators is currently limited to major teaching hospitals, creating nationwide disparities in terms of access. High acquisition and maintenance costs of the AI-integrated systems, in addition to limited funding for simulation faculty and equipment, restrict the scalability of a nationwide programme. Integration of AI into existing NHS education budgets will first require a clear demonstration of cost-effectiveness and improved patient safety outcomes if it is to align with the NHS Long Term Workforce Plan.

Future Directions

Looking ahead, the UK should prioritise the establishment of a national AI-in-Surgery Integration Committee to coordinate validation, standardisation and equitable access across training programmes. This would contain consultant surgeons, NHS managers, AI developers and surgeons in training to allow for a combined holistic approach. Embedding AI-derived metrics within the ISCP logbook and portfolio would allow objective and longitudinal tracking of trainee progression, enabling consistent benchmarking across deaneries.

Future research must move beyond feasibility studies and incorporate more rigorous methodologies, including multi-centre prospective cohort studies and, where appropriate, randomised controlled trials comparing AI-enhanced training with conventional methods. Such studies should evaluate specific educational outcomes such as skill retention, transfer of simulator performance to real operative settings, operative efficiency, error reduction and patient-safety outcomes. In parallel, qualitative work should examine how AI influences learning behaviour, feedback quality and trainee autonomy.

Achieving these goals will require coordinated partnerships between NHS trusts, simulation companies and academic institutions, including the RCS, to facilitate the development of large, secure and General Data Protection Regulation (GDPR)-compliant datasets that support robust evaluation and scalable national implementation.

## Conclusions

This review highlights that AI technologies are increasingly present within UK surgical training, supported by RCS initiatives and a growing number of early pilot studies. The included evidence demonstrates the feasibility of AI-enhanced tools, such as motion-tracking assessment systems, adaptive VR and AR simulation, and robotic performance analytics, indicating positive trainee engagement. However, the current evidence base remains limited by small pilot cohorts, a lack of standardised metrics, and an absence of large studies demonstrating clear improvement in operative performance or patient outcomes. This gap underscores the need for more robust and generalisable research before AI can be fully integrated into national training pathways. From a policy perspective, coordinated national leadership will be critical to ensure consistent and equitable adoption of AI across the NHS throughout all the deaneries within the UK. Actionable steps include the establishment of a national AI-in-surgical-training Integration committee, the development of standardised performance AI frameworks aligned with ISCP requirements, secured funding streams for simulation and digital training infrastructure, and the creation of GDPR-compliant national datasets to support algorithm validation. Embedding AI-derived metrics within e-portfolios and assessment tools would further promote objective, longitudinal evaluation of trainee progression.

Important limitations remain in the current research landscape. The predominance of single-centre feasibility work restricts generalisability, and there is a clear need for multi-centre prospective studies, randomised controlled trials, and long-term follow-up to evaluate outcomes such as skill retention, operative efficiency, patient safety and transfer of simulated skills to the operating theatre. Ethical issues such as algorithm transparency, bias, data governance and impacts on trainee autonomy. This must also be addressed within UK regulatory frameworks such as the MHRA SaMD/AIMD guidance and NHS AI governance standards. In summary, AI should not replace traditional mentorship or operative experience. It should serve as a complementary tool that supports objective assessment, enhances feedback, and future-proofs the surgical workforce. A coordinated national strategy, supported by rigorous research and aligned policy, will be essential for the UK to transition from isolated pilot projects to a cohesive, evidence-based integration of AI into surgical education.
